# Collating the knowledge base for core outcome set development: developing and appraising the search strategy for a systematic review

**DOI:** 10.1186/s12874-015-0019-9

**Published:** 2015-03-29

**Authors:** Elizabeth Gargon, Paula R Williamson, Mike Clarke

**Affiliations:** Department of Biostatistics, University of Liverpool, Liverpool, UK; All Ireland Hub for Trials Methodology Research, Queen’s University Belfast, Belfast, UK

**Keywords:** Core outcome set, Database, Resources, Systematic review, Medical research

## Abstract

**Background:**

The COMET (Core Outcome Measures in Effectiveness Trials) Initiative is developing a publicly accessible online resource to collate the knowledge base for core outcome set development (COS) and the applied work from different health conditions. Ensuring that the database is as comprehensive as possible and keeping it up to date are key to its value for users. This requires the development and application of an optimal, multi-faceted search strategy to identify relevant material. This paper describes the challenges of designing and implementing such a search, outlining the development of the search strategy for studies of COS development, and, in turn, the process for establishing a database of COS.

**Methods:**

We investigated the performance characteristics of this strategy including sensitivity, precision and numbers needed to read. We compared the contribution of databases towards identifying included studies to identify the best combination of methods to retrieve all included studies.

**Results:**

Recall of the search strategies ranged from 4% to 87%, and precision from 0.77% to 1.13%. MEDLINE performed best in terms of recall, retrieving 216 (87%) of the 250 included records, followed by Scopus (44%). The Cochrane Methodology Register found just 4% of the included records. MEDLINE was also the database with the highest precision. The number needed to read varied between 89 (MEDLINE) and 130 (SCOPUS).

**Conclusions:**

We found that two databases and hand searching were required to locate all of the studies in this review. MEDLINE alone retrieved 87% of the included studies, but actually 97% of the included studies were indexed on MEDLINE. The Cochrane Methodology Register did not contribute any records that were not found in the other databases, and will not be included in our future searches to identify studies developing COS. SCOPUS had the lowest precision rate (0.77) and highest number needed to read (130). In future COMET searches for COS a balance needs to be struck between the work involved in screening large numbers of records, the frequency of the searching and the likelihood that eligible studies will be identified by means other than the database searches.

**Electronic supplementary material:**

The online version of this article (doi:10.1186/s12874-015-0019-9) contains supplementary material, which is available to authorized users.

## Background

Differences in outcomes and the difficulties caused by heterogeneity in outcome measurement are well known [1]. There is inconsistency in outcome measurement in clinical trials, and not all trials of a specific condition provide data on important outcomes. Furthermore, there are often differences in how outcomes are defined and measured making it difficult, sometimes impossible, to synthesise the results and apply them in a meaningful way. Alongside this inconsistency in the measurement of outcomes, outcome reporting bias adds further to the problems faced by users of research [2]. Publication of complete trial results is important to practitioners, consumers, and policy makers who wish to make well-informed decisions about health and social care. However, this does not always happen and outcomes that are statistically significant are more likely to be fully reported [3]. Furthermore, in a sensitivity analysis that sought to account for outcome reporting bias in reviews with a statistically significant result, it was found that 19% would not have remained significant had all studies contributed to the analyses and 26% had overestimated the treatment effect by more than 20% [4]. These issues of inconsistency and outcome reporting bias could be addressed with the development and application of agreed standardised sets of outcomes, known as COS, that should be measured and reported in all trials for a specific clinical area [5].

The COMET (Core Outcome Measures in Effectiveness Trials) Initiative (www.comet-initiative.org) brings together people interested in the development and application of core outcome sets (COS). COMET aims to collate and stimulate relevant resources, both applied and methodological, to facilitate exchange of ideas and information, and to foster methodological research in this area. The importance of COS is increasingly recognised by research funders. For instance, the National Institute for Health Research’s Health Technology Assessment programme in the UK, the Health Research Board in Ireland and the charity Arthritis Research UK, are all highlighting this to researchers seeking funding for new studies. However, the identification of existing COS is not easy.

As part of the COMET Initiative, we are developing a publicly accessible internet-based resource to collate the knowledge base for COS development and the applied work that has already been done according to disease area. This will be a useful resource for trial funders to refer to, for researchers to see what work has been done in their area of interest and for research funders wishing to avoid unnecessary duplication of effort when supporting new activities. It will include planned and ongoing work, as well as published accounts of COS development. Prior to the completion of the systematic review outlined here, 120 relevant studies had been identified through known research networks, but creating a comprehensive database and keeping the database up to date is key to its value for users and requires a more structured and transparent approach than the ad hoc inclusion of studies as they come to light. This requires the development and application of an optimal, multi-faceted search strategy to identify work related to the development of COS. This paper outlines the methodological approach taken to develop the search strategy for this systematic review of studies of COS development [6], and, in turn, to establish a comprehensive database of COS.

We developed an appropriate search strategy to identify as many relevant studies as possible within the available resources, and then investigated the performance characteristics of this strategy. We aimed to compare the contribution of databases towards identifying included studies, and identify the best combination of methods to retrieve all included studies.

## Methods

### Developing an appropriate search strategy

**Figure 1:**
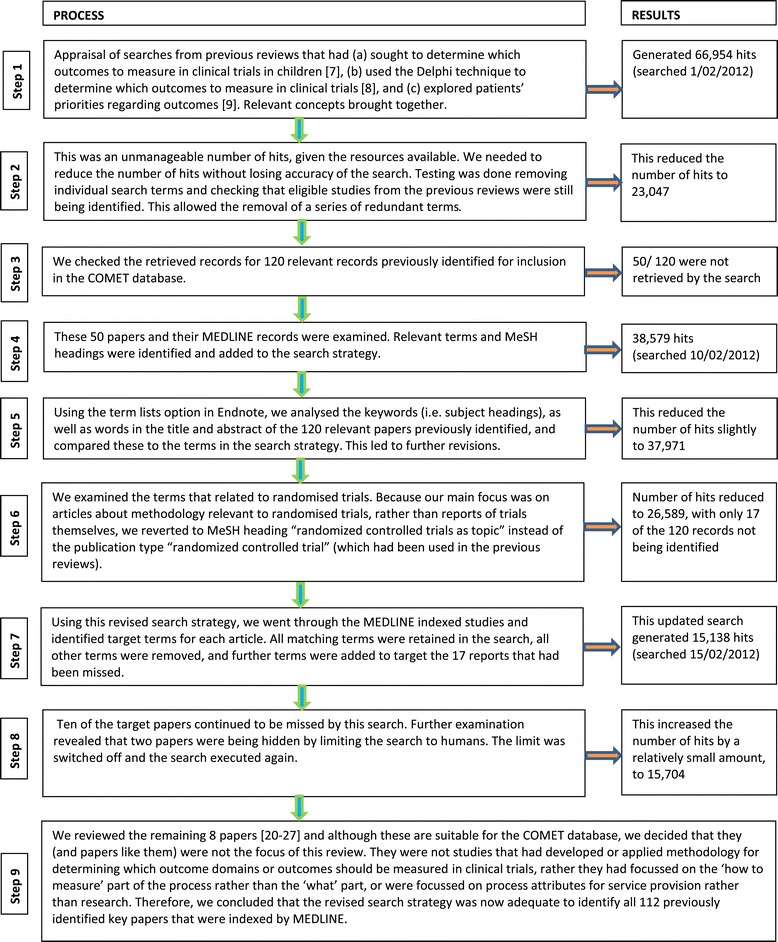
**Developing an appropriate search strategy [** 20 **-** 27 **].**

We developed a multi-faceted search strategy to search electronic databases using a combination of text words and index terms, adapting the search strategy as appropriate for each database. Studies relating to participants of any age, with any health condition in any setting and assessing the effect of any intervention were eligible for inclusion. We used an iterative process to develop this search, firstly for MEDLINE via Ovid. This was built on an appraisal of three searches: a previous review of studies that aimed to determine which outcomes to measure in clinical trials in children [7]; a review of studies using the Delphi technique to determine which outcomes to measure in clinical trials [8], and a review of studies addressing patients’ priorities regarding outcomes [9]. These three searches were merged into a single strategy and adapted for use in MEDLINE via Ovid. This combined set of search terms generated 66954 hits (searched February 2012), which we deemed an unmanageable number of hits. We therefore needed to reduce the number of hits whilst retaining the precision of the search. This process is described in Figure 1. The search was then modified for use in the other electronic databases that we considered searching for this study.

The final search strategy is given in Additional file 1 and combines three concepts of search terms, covering ‘randomised trial/systematic review’, ‘methodology’ and ‘outcomes’. All terms in each concept were combined with the Boolean operator OR and the three concepts were then combined using the Boolean operator AND. Key terms were also targeted, and these terms were combined with the Boolean operator OR. Truncation and wildcards were used to improve the sensitivity of the search, account for spelling variations and to identify different derivations of search terms.

### Confirming the need for such a large search

As described in Figure 1, the final search was still retrieving a large number of records and our next step was to determine if this was because there were many eligible papers for the systematic review or because we were continuing to retrieve an overwhelming proportion of irrelevant material. We also wished to develop an estimate of the likely number of COS in the literature. Therefore, we examined the potential relevance of 1% of the retrieved records. A random number generator (R) was used to select records, and their titles and abstracts were read to identify potentially relevant studies.

### Electronic databases

The high yield of eligible studies from MEDLINE and evidence that no single database is likely to be sufficient for identifying research across all areas of health care [10-12], led to a variety of electronic databases being considered for searching:

1. MEDLINE via Ovid
2. The Cochrane Library (excluding categories ‘Trials’ and ‘Cochrane Groups’)
3. Cumulative Index of Nursing and Allied Health Literature (CINAHL) plus
4. Scopus

MEDLINE focuses on biomedical journal literature, CINAHL on nursing and allied health literature, and Scopus covers medical and scientific literature, so we thought each had a sufficiently different focus to consider including in the search. Previous work that has been done to consider the coverage between CINAHL and Scopus to determine whether Scopus alone provides sufficient coverage of the literature concluded that only partial duplicate coverage of nursing and allied health literature was offered by CINAHL [10]. While SCOPUS’s significantly larger coverage may offer many unique titles in these subject areas, it is not possible to say that these titles would be an adequate substitute for CINAHL’s coverage of this literature. As its relevance to COS work was not yet known, it warranted further exploration. EMBASE was also considered for inclusion. We decided to include SCOPUS as opposed to EMBASE as it is a larger database and offers more coverage of scientific, technical, medical and social science literature [3]. Furthermore, SCOPUS indexes all EMBASE journals. The relevant modified search strategy was applied to each of these databases and each was considered in turn for suitability for inclusion in the final strategy.

### Hand searching

In addition to the electronic database searching, we completed a range of hand searching activities, in keeping with research evidence showing the added benefits of hand searching alongside electronic searching [13]. We identified and reviewed funded projects that included the development of a COS, including National Institute for Health Research (NIHR) programme grant scheme reports and Health Technology Assessment (HTA) reports; searched for known key authors and citations to key papers, for example, the work of the OMERACT (Outcome Measures in Rheumatology) group; examined references cited in eligible studies and in other studies that referred to or used a COS. We also contacted the Cochrane Review Groups (CRGs) to request information on COS that they were aware of (see Kirkham et al. [14] for full details of the methods).

### Sensitivity, precision and numbers needed to read (NNR)

We recorded whether each included study was:

i. retrieved by the search strategy developed for each database
ii. indexed on each database (regardless of whether or not it was retrieved by the search of the database)

The recall (or sensitivity), precision and numbers needed to read (NNR) for the final searches in each of the databases were calculated using the following definitions [15]:

- Recall (%) = 100 × (Number of included records retrieved/Total number of included records)
- Precision (%) = 100 × (Number of included records retrieved/Total number of records retrieved)
- NNR = 1/precision
- Unique yield = number of studies retrieved only by this database
- In addition, sensitivity*precision was calculated to allow a balance between sensitivity and precision to be assessed.

## Results

The results of the search strategy development process are shown in Figure 1. When one author examined 1% (n = 157) of the large number of retrieved records (n = 15,704), 30 (19%) were identified as being potentially relevant, including three that we had previously identified as eligible studies. It was determined that 8 of the other 27 records were eligible following an assessment of their full papers. This confirmed that the search strategy was identifying relevant studies, that the likely yield of such studies was likely to be high and that a formal, systematic review was necessary to identify papers that were not yet known to COMET if we were to create a comprehensive resource that others could use to determine whether or not a COS had already been developed in an area of interest to them.

The search strategy was modified as appropriate for each database. The combined results (searched 29 May 2012) generated a total of 47,225 records (MEDLINE n = 14,520, Cochrane Library n = 4122, CINAHL n = 16,700, Scopus n = 11,883), which fell to 37,132 after removal of duplicates (duplicates accounted for approximately 22% of total). We therefore needed to consider each of the additional databases that we were planning to search more carefully to estimate their likely added yield over MEDLINE. For example, The Cochrane Library is a collection of six databases that contain different types of research: Cochrane Database of Systematic Reviews; Cochrane Central Register of Controlled Trials; Cochrane Methodology Register; Database of Abstracts of Reviews of Effects; Heath Technology Assessment Database, and NHS Economic Evaluation Database. Each of these has a different focus, with the Cochrane Methodology Register being most relevant to this project, but it should be noted that work on the development and maintenance of the Register was suspended by the UK Cochrane Centre in May 2012 and it has not been updated since July 2012. The Cochrane Methodology Register includes articles that are relevant to the methods for systematic reviews, trials and other evaluations of health and social care and, as such, would be the most relevant component of The Cochrane Library for reports on the development of COS. With this in mind, the search strategy that was developed for The Cochrane Library as a whole was then limited to the Cochrane Methodology Register. It is recognised that The Cochrane Methodology Register has a unique controlled vocabulary but a decision was made to use the generic approach typically used when searching the whole Cochrane Library as this had already been developed for this search. For Scopus and CINAHL, two of the authors (EG and PW) independently reviewed a sample of abstracts from each. Fifty abstracts from Scopus yielded three eligible records that were not retrieved by MEDLINE or CINAHL. For CINAHL, a review of 100 abstracts excluded 91 as ineligible based on the abstract and the remaining nine potentially eligible studies were all identified by MEDLINE, Scopus or both. As a consequence, it was agreed that CINAHL would not be used, at least in this first round for the systematic review.

Therefore, the following electronic databases were searched (August 2013, see Additional file 1):

1. MEDLINE via Ovid
2. SCOPUS
3. Cochrane Methodology Register

This updated search identified 34,398 potentially relevant records, all of which were checked and 220 eligible records were found (the process for selecting studies for inclusion in the review is fully described in the main paper [6]). Fifty-nine (27%) were already known to us, so the search identified an additional 161 records. In addition to the database search, 30 additional records that had not been previously identified were deemed eligible after being identified through hand searching. A full list of the 250 included records is provided as a supplementary table (Additional file 1: Table S1) in the main paper [6]. Recall of the search strategies ranged from 4% to 87%, and precision from 0.77% to 1.13% (Table 1). MEDLINE via Ovid performed best in terms of recall, retrieving 216 (87%) of the 250 included records, followed by Scopus (44%). The search of the Cochrane Methodology Register identified just 4% of the included records, and all of these were found in at least one of the other databases. MEDLINE via Ovid was also the database with the highest precision. The number needed to read varied between 89 (MEDLINE via OVID) and 130 (SCOPUS). If our searches had been limited to MEDLINE alone, only 3 included records unique to SCOPUS would not have been retrieved.

**Table 1:** **Recall, precision and NNR for each strategy**

**Database**	**Number of records retrieved**	**Number of included records**	**Unique yield**	**Recall (%) (n = 250)**	**Precision (%)**	**Number needed to read (NNR)**	**Sensitivity × Precision**
MEDLINE via OVID	19058	216	109	87	1.13	89	98.31
SCOPUS	14258	109	3	44	0.77	130	33.88
Cochrane Methodology Register	1082	9	0	4	0.83	121	3.32

Through examining references cited in eligible studies and in other studies that referred to or used a COS, 30 additional records were identified and included in the systematic review. No additional studies were identified through the survey of Cochrane Review Groups. Of the 30 records identified via hand searching and not retrieved by the search strategy, we found that two were not indexed on any database, 25 were indexed on both Medline and Scopus, two on Scopus only, and one was in all three databases. On closer inspection, we found that the reasons for non-retrieval of the 28 studies by the database searches was the wide variety of free text and index terms used in their records. As has been shown in other contexts, modifications to the search to retrieve all these records would have produced searches with unmanageably large numbers of records [16]. Furthermore, the absence of two of the reports from the searched databases highlights that even such extensive searching would not have retrieved all the studies that we identified. However, one of these was not a journal article, and the other was an editorial which provided additional methodological information on a study that had been retrieved in its own right.

## Discussion

COS are increasingly recognised as important for the design, conduct and reporting of randomised trials, systematic reviews and other forms of research. However, as we have shown in this paper, the development of a search strategy to identify them is challenging. A search for COS in any specific area could combine the approach we have taken with search terms for specific conditions or interventions, but it is still likely to require a large number of records to be checked to identify the few that are eligible. We hope therefore that our comprehensive approach to searching a major database such as MEDLINE for all reports of studies developing COS, regardless of the setting, and the subsequent inclusion of identified studies in the COMET database will make it much easier for researchers in the future. This is akin to the work of The Cochrane Collaboration in identifying reports of randomised trials regardless of topic area for inclusion in the Cochrane Central Register of Controlled Trials [17,18].

In undertaking this comprehensive approach to identifying COS, we encountered similar challenges to those faced by healthcare researchers in the past. For example, variability in the use of free text terms and index terms on reports of randomised trials of portal vein infusion chemotherapy in colorectal cancer meant that a search to identify all articles that had been identified for a systematic review would have had to rely solely on the terms for colorectal cancer, which retrieved 18,450 records [16]. On a larger scale, when The Cochrane Collaboration was established in 1993, although tens of thousands of reports of randomised trials could be found easily in MEDLINE, there were many more than had not been appropriately indexed and could not be found so easily. The development of highly sensitive search strategies and subsequent work within the Collaboration to find these “hidden” reports, led to the identification an additional 70,000 records that were re-tagged as randomised controlled trials or controlled clinical trials in MEDLINE and can now be found using those terms [17,18]. In a similar way, our comprehensive searching for COS regardless of any particular healthcare condition and their inclusion in the COMET database makes it much easier for users to access these studies.

Although work on the development of COS goes back at least 30 years [19], the term itself has not been widely used until relatively recently and there are currently no MeSH headings in MEDLINE or index terms in other bibliographic databases for identifying COS papers, and they do not appear to be categorised consistently across different databases. Furthermore, no single database specialises in this type of methodological research and it is likely to be found across a wide range of literature. For example, MEDLINE and EMBASE focus on biomedical journal literature, CINAHL on nursing and allied health literature, and Scopus covers medical and scientific literature. Each database has a different focus but each could include studies of the development of COS. Furthermore, no single database is likely to be adequate. For instance, a comparison of the coverage between CINAHL and Scopus to determine whether Scopus alone provides sufficient coverage of the literature found that Scopus can only partially duplicate the coverage of nursing and allied health literature offered by CINAHL [10]. Other comparisons, of other combinations of databases, have also shown how systematic reviews are likely to benefit from searching for potentially eligible studies in several databases [11,12].

The search strategy we developed for COS has been designed to be highly sensitive, so that as many potentially relevant studies as possible will be retrieved. The final effective search strategy combines keywords, index terms and free-text terms and phrases, using combinations of Boolean operators. As no MeSH headings or index terms currently exist for COS papers, and these papers do not appear to be indexed in a consistent way, key search terms were also targeted and no limits were applied to the search. A consequence of a highly sensitive search is usually that a large number of irrelevant records will be retrieved, the majority of which will likely not meet the inclusion criteria for the review, and this appears to be the case here.

We found that two databases and hand searching were required to locate all of the studies that we were able to include in this review. MEDLINE via Ovid alone retrieved 87% of the included studies, but actually 97% of the included studies were indexed on MEDLINE. The search of the Cochrane Methodology Register did not identify any records that were not found in the other databases. We identified retrospectively that the search in The Cochrane Library used an implied ‘AND’ in some of the lines which could affect the sensitivity and precision of this search. Given that its development and maintenance was suspended in July 2012, it will not be included in our future searches to identify studies developing COS. However, this decision will be reviewed and the search strategy evaluated should work on the Register resume. SCOPUS had the lowest precision rate (0.77) and highest number needed to read (130), which is a particularly high number of records to check in order to find one relevant record. Therefore, with such a low unique yield from SCOPUS (3 studies identified with the search strategy, increasing to 5 actually indexed), it might not be worth searching SCOPUS, given the low precision and high NNR. Furthermore, the decision to search SCOPUS may have increased the de-duplication burden due to the lack of flexibility in its interface, for example de-duplicating against MEDLINE within OVID. This could be an added advantage of searching EMBASE and may be considered in any future updates to this search.

## Conclusions

In considering how this analysis might inform future decisions about the COMET searches for COS, a balance needs to be struck between the work involved in screening large numbers of records, the frequency of the searching and the likelihood that eligible studies will be identified by means other than the database searches. One possibility is that the comprehensive searching is limited to MEDLINE, perhaps with further appraisal of the unretrieved records to seek ways to modify the search to target these papers. The search might also continue to include SCOPUS, but consideration could be given to other databases that were not included in this project, such as EMBASE. In keeping with the research evidence, it would seem there is an added benefit to hand searching, so this should accompany any electronic searching in the future. This would help to identify relevant studies that are missed by the database searching, either because they are not indexed in the databases or are indexed but cannot be retrieved without using a search strategy that would yield an unmanageable number of records to check. To keep the database current we aim to complete an annual search of MEDLINE and SCOPUS as a minimum. Finally, to supplement this annual search, the COMET database will continue to be populated with studies, both ongoing and completed, that are identified by ad hoc means and sent directly to the COMET team.

## Additional file

Additional file 1: Table S1. Search terms.
