# Supplementary material for: Collating the knowledge base for core outcome set development: developing and appraising the search strategy for a systematic review
Source: BMC Med Res Methodol. 2015 Mar 29;15:26. doi: 10.1186/s12874-015-0019-9 (PMC4395975; doi:10.1186/s12874-015-0019-9)
Supplement: Additional file 1: Table S1. — Search terms. [file 12874_2015_19_MOESM1_ESM.pdf]

Table 1: Search terms

	Search terms for MEDLINE	Number of hits (searched August 2013)
	**Randomised trial and systematic review terms**	
1	Health Services/ut [Utilization]	5742
2	registries/	45459
3	systematic review.mp.	28277
4	structured review.ti.	108
5	evidence based medicine.ab.	4843
6	exp Clinical Trials as Topic/	258536
7	clinical trial$.ab.	149831
8	randomised controlled trial$.ti,ab.	16321
9	randomised trial$.ti,ab.	9858
10	1 or 2 or 3 or 4 or 5 or 6 or 7 or 8 or 9	444510
	**Methodology terms**	
11	workgroup$.mp.	757
12	standard$ outcome$.mp.	519
13	Practice Guideline/	16919
14	clinical database.mp.	908
15	patient important outcome$.mp.	78
16	(standard$ adj3 reporting).mp.	1999
17	congresses.pt.	57907
18	Delphi Technique/	2274
19	(recommend$ adj3 outcome$).mp.	1062
20	consensus development conference.pt.	8085
21	outcome$ reporting.mp.	267
22	priorit$ symptom$.mp.	23
23	(task force adj3 outcome$).mp.	49
24	appropriate outcome$.mp.	338
25	research design/	67019
26	endpoint determination/	3416
27	consensus development conference/	8085
28	patient participation/	16143
29	consensus.mp.	93957

30	workshop.mp.	17486
31	Consensus Development Conferences, NIH as Topic/	314
32	focus groups/	13711
33	11 or 12 or 13 or 14 or 15 or 16 or 17 or 18 or 19 or 20 or 21 or 22 or 23 or 24 or 25 or 26 or 27 or 28 or 29 or 30 or 31 or 32	279051
	**Outcome terms**	
34	outcome$.mp.	1156289
35	end point$.mp.	32706
36	(core adj3 set).mp.	1510
37	treatment emergent problem$.mp.	1
38	exp outcome Assessment Health Care/	593962
39	Treatment Outcome/	535004
40	Quality of Life/	101029
41	34 or 35 or 36 or 37 or 38 or 39 or 40	1256710
	**Key terms targeted**	
42	clinical-study design.mp.	82
43	patient$ perspective$.ti.	1387
44	outcome$.mp. and delphi.ti.	153
45	(outcome$ and delphi).ab.	624
46	(perspective$ adj3 outcome$).ti.	102
47	core outcome$.ti,ab.	121
48	core set$.ti,ab.	1124
49	clinical trial design$.ti.	355
50	design$ clinical trial$.ti.	72
51	(consensus and outcome$).ti.	133
52	42 or 43 or 44 or 45 or 46 or 47 or 48 or 49 or 50 or 51	3931
53	10 and 33 and 41	12607
54	52 or 53	16079
**Search terms for SCOPUS**		
((((INDEXTERMS(registries)) OR (INDEXTERMS(clinical trials as topic)) OR (ABS("evidence based medicine")) OR (ABS("clinical trial*")) OR (INDEXTERMS("Health Services Utilization")) OR (TITLE-ABS-KEY("SYSTEMATIC REVIEW")) OR (TITLE("structured review"))) OR (TITLE OR ABS("randomised controlled trial*")) OR (TITLE OR ABS (randomised trial*))) AND (((TITLE-ABS-KEY(workgroup*)) OR (TITLE-ABS-KEY(standard* outcome*)) OR (INDEXTERMS(practice guideline)) OR (TITLE-ABS-KEY("clinical database")) OR (TITLE-ABS-KEY("patient important outcome*")) OR (TITLE-ABS-KEY("standard* outcome*")) OR (INDEXTERMS(delphi technique))) OR ((TITLE-ABS-KEY(recommend* W/3 outcome*)) OR (TITLE-ABS-KEY(standard* W/3 reporting*)) OR (TITLE-ABS-KEY(task force W/3 outcome*)) OR (TITLE-ABS-KEY("appropriate		12286

outcome*")) OR (TITLE-ABS-KEY("outcome* reporting")) OR (TITLE-ABS-KEY("priorit* symptom*")) OR (INDEXTERMS(focus group)) (INDEXTERMS(research design))) OR ((INDEXTERMS(endpoint determination)) OR (INDEXTERMS(consensus development conference)) OR (INDEXTERMS(patient participation)) OR (TITLE-ABS-KEY(consensus)) OR (TITLE-ABS-KEY(workshop)))) AND 74) OR (((TITLE("design* clinical trials")) OR (TITLE(consensus AND outcome*)) OR (TITLE-ABS-KEY("clinical-study design")) OR (TITLE("patient* perspective*")) OR (ABS(outcome* AND delphi)) OR (TITLE(outcome* AND delphi)) OR (TITLE(perspective* W/3 outcome*)) OR (ABS("core outcome*") OR TITLE("core outcome*"))) OR ((ABS("core set*") OR TITLE("core set*")) OR (TITLE("clinical trial design*"))))		
**Search terms for The Cochrane Library**		
	**Randomised trial and systematic review terms**	
#1	(clinical trial*):ab	58990
#2	MeSH descriptor Health services	750
#3	MeSH descriptor registries	604
#4	(systematic review):ti,ab,kw	13816
#5	(structured review):ti	6890
#6	(evidence based medicine):ab	681
#7	MeSH descriptor Clinical Trials as Topic explode all trees	1117
#8	(randomised controlled trial):ti or (randomised controlled trial):ab	79593
#9	(randomised trial*):ti,ab,kw	158334
#10	(**#1** OR **#2** OR **#3** OR **#4** OR **#5** OR **#6** OR **#7** OR **#8 OR #9**)	126425
	**Methodology terms**	
#11	(workgroup*):ti,ab,kw	24
#12	MeSH descriptor Practice Guideline	1221
#13	(patient important outcome*):ti,ab,kw	3977
#14	(clinical database):ti,ab,kw	2335
#15	standard* NEAR/3 reporting	2310
#16	(congresses):pt	45
#17	MeSH descriptor Delphi Technique explode all trees	33
#18	recommend* NEAR/3 outcome	309
#19	(consensus development conference):pt	4
#20	(priorit* symptom*):ti,ab,kw	964
#21	(task force NEAR/3 outcome*):ti,ab,kw	4
#22	(appropriate outcome*):ti,ab,kw	2528
#23	MeSH descriptor Focus Groups explode all trees	232

#24	MeSH descriptor Research Design	1811
#25	MeSH descriptor endpoint determination	61
#26	MeSH descriptor consensus development conference	570
#27	MeSH descriptor patient participation	354
#28	(consensus):ti,ab,kw	2049
#29	(workshop):ti,ab,kw	955
#38	"standard outcome*":ti,ab,kw	27
#39	"outcome* reporting":ti,ab,kw	191
#37	(**#11 OR #12** OR **#13** OR **#14** OR **#15** OR **#16** OR **#17** OR **#18** OR #19 OR **#20** OR **#21** OR **#22** OR **#23** OR **#24** OR **#25** OR **#26** OR **#27** OR **#28** OR #29 OR **#38** OR **#39**)	14699
	**Outcome terms**	
#30	(outcome*):ti,ab,kw	143278
#31	(end point*):ti,ab,kw	12764
#32	(core NEAR/3 set):ti,ab,kw	82
#33	(treatment emergent problem*):ti,ab,kw	25
#34	MeSH descriptor Outcome Assessment (Health Care) explode all trees	81711
#35	MeSH descriptor Treatment Outcome	1957
#36	MeSH descriptor quality of life	1517
#40	(**#30** OR **#31** OR **#32** OR **#33** OR **#34** OR **#35** OR **#36**)	151831
#41	(#10 AND #37 AND #40)	7096
	**Key terms targeted**	
#42	(design* clinical trials):ti	541
#43	(clinical-study design):ti,ab,kw	2081
#44	(patient* perspective*):ti	161
#45	(outcome*):ti and (delphi):ti	0
#46	(outcome*):ab and (delphi):ab	62
#47	(perspective* NEAR/3 outcome*):ti	5
#48	(core outcome*):ti or (core outcome*):ab	616
#49	(core set):ti or (core set):ab	383
#50	(clinical trial design*):ti	541
#51	(outcome):ti and (consensus):ti	8
#52	(#42 OR #43 OR #44 OR #45 OR #46 OR #47 OR #48 OR #49 OR #50 OR #51)	3674

#53	(#41 OR #52)	10572
#54	(#53)<br>*In Methods Studies*	1082
